# Wheel-Running Modestly Promotes Functional Recovery after a Unilateral Cortical Lesion in Rats

**DOI:** 10.1155/2005/105341

**Published:** 2005-08-04

**Authors:** Xiurong Zhao, Jaroslaw Aronowski, Shi-Jie Liu, Timothy Schallert, Jie Zhang, Roger Strong, Zhi-Shuo Ou, Theresa Nguyen, James C. Grotta

**Affiliations:** ^1^Department of NeurologyUniversity of TexasMedical SchoolHoustonTX 77030USA; ^2^University of Texas at AustinInstitute for NeuroscienceAustinTX 78712USA

**Keywords:** NMDA, excitotoxicity, wheel-running, sensorimotor function, synaptophysin, cortical lesion, stroke, cerebral ischemia, recovery, exercise

## Abstract

*Background:* We aimed to determine whether early or delayed wheel-running (W) after a cortical lesion in rats influences functional recovery and protein expression involving synaptic plasticity.

*Methods:* 57 rats were arranged in 4 groups: (1) Sham, (2) NMDA, (3) W-24 h or (4) W-72 h (W-24 h and W-72 h means wheel-running for 14 days starting day 1 or day 3 after NMDA lesion). NMDA produced a standardized lesion in the unilateral sensorimotor cortex and detectable behavioral deficits. Synaptogenesis was measured by immunohistochemistry.

*Results:* Wheel-running starting after 24 h had no detectable effect, but it significantly speeded functional recovery when delayed to after 72 h. These results were in accordance with a marker linked to synaptogenesis.

*Conclusion:* Wheel-running starting 3 days, but not 1 day, after an NMDA lesion is associated with improved functional recovery.

## 1. Introduction

It is a common practice among clinical stroke centers to provide physical/occupational therapy and rehabilitation programs to patients shortly after stroke onset. Procedures that encourage mobilization of the patient may reduce secondary complications and mortality, and may promote long-term functional outcome after stroke onset [1]. Recent studies have suggested that the intensity of this training, as well as the time point at which it is implemented, could have profound effects on outcome [2–4]. Early initiated treadmill training starting 24 h after focal brain ischemia promoted neurological function recovery in rats, but this was not seen when the treadmill training program was started one week later [5,6]. In other studies, delayed training starting 8 days after striatal hemorrhage was shown to rescue neurons from delayed chronic degeneration [7]. Therefore, the optimal time window for therapeutic intervention is still not clearly evident.

Many clinical and experimental studies have shown that exercise training can promote adaptive plasticity in the healthy brain as well as after a brain lesion [4,8–13]. The precise anatomical, cellular, and molecular mechanisms of functional recovery following stroke remain unknown. Animal studies have suggested that exercise training may promote central neuronal plasticity [12]. Local neuronal sprouting and synaptic reorganization may occur and contribute to functional recovery [14–16]. Exercise training can induce angiogenesis [4] and release of neurotropins [17].

Some reports, however, have indicated that intense early training might, under certain conditions, have

ISSN 0953-4180/05/$17.00 © 2005 – IOS Press and the authors. All rights reserved negative effects [18–20]. For example, immobilization of the forelimb ipsilateral to the lesion immediately after frontal cortical focal stroke, and consequent "forced overuse" of the affected forelimb, can slow recovery [21–23]. Researchers have also found that overuse of the affected forelimb can increase tissue loss around a focal cortical injury [18,19].

In order to determine if early exercise training after a cortical lesion is beneficial, harmful, or neutral, we studied the effect of motor-training by a running wheel started at two different intervals soon after an NMDA insult that produces a highly standardized and localized cortical lesion that predictably results in forelimb dysfunction on the contralateral side. In order to minimize the factor of stress on outcome, we placed the rats in a running-wheel with an attached cage that ensured food and water availability ad libitum, and rest and exercise voluntarily. We compared morphologic and functional outcome in control rats housed in standard cages with that of rats freely doing wheel-running,

We used synaptophysin immunoactivity to measure synaptogenesis. Synaptophysin, a presynaptic vesicle protein found in virtually all nerve terminals, is a protein marker of axonal sprouting and synaptogenesis. The levels of synaptophysin within the terminal remain constant as a result of recycling of the vesicle materials and have been used to quantitate numbers of terminals during neuroanatomical remodeling and neural development.

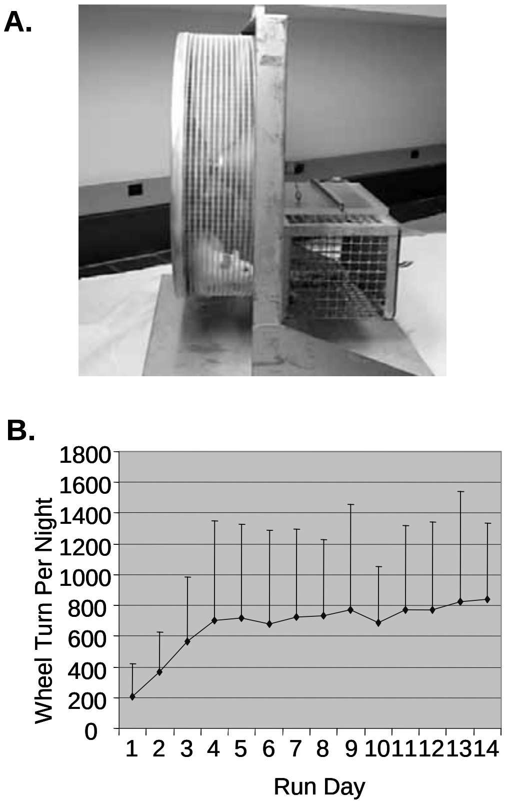

Fig. 1. Photograph of the running wheel (A) and daily running log on days 1 to 14 after NMDA injection in the W-24 h and W-72 h groups (B). There were no significant differences between groups, so we pooled the data from all the rats. The data are expressed as mean $\pm$ SD.

## 2. Materials and methods

### 2.1. Subjects

57 male Sprague-Dawley (Harlan Sprague Dawley) rats, weighing 250 to 300 g at the time of surgery, were used. Animals were kept on a 12:12 h light: dark cycle and allowed food and water ad libitum. Animals were tamed by gentle handling twice daily for one week before inclusion in the experiment.

Before the experiments, the rats were first trained and selected for wheel-running by placement in a cage with the wheel (Fig. 1A) for 2 hours. The rats had voluntary access to the wheel as well as to food and water. If they voluntarily used the wheel for more than 40 turns, they were included into our study. Non-runners were presented with this selection step twice. After wheel-runners were selected, they were pre-trained and pre-tested on behavior tests including footfault, cylinder, and vibrissa-evoked forelimb placing. Only rats with less than 20% footfaults, greater than 50% simultaneous placing of both forelimbs on the cylinder test, and greater than 90% forelimb placing after whisker stimulation were included for further study.

### 2.2. Animal surgery

Rats were anesthetized with 0.35 g/kg chloral hydrate, ip, and placed in a head holder. Lesions were made by stereotaxic infusion of 100 nmol NMDA (Sigma, St. Louis, MO) in 1.0 $\mu$l of saline via a 30 G stainless steel cannula connected via Teflon tubing to a 5-$\mu$l glass Hamilton microsyringe mounted in a infusion pump. The delivery speed was controlled at a constant infusion speed of 1.0 $\mu$l in 60 min. After the injection was complete, the cannula was left in place for 20 min to allow proper diffusion. The stereotaxic coordinates used for intracerebral injection were 3.5 mm lateral, $+$ 0.5 mm from the bregma, and 1.2 mm deep from the dura ventrally. This lesion, always on the left, included a portion of the right forelimb sensorimotor cortex.

### 2.3. Housing and group arrangements

Rats were housed in standard cages, two rats per cage. Those assigned to the wheel-running group (see below) were placed in a cage with an opening to an attached wheel (see Fig. 1A) to provide rats with ad libitum access to food and water, and rest and activity voluntarily from 5 pm until 9 am the next day in the dark cycle. During the day the rats were put back into their home cage. The number of wheel turns was recorded when the rat was returned to its home cage. These steps were repeated for 14 nights beginning 1 or 3 days after surgery. Therefore, there were four groups:

**SHAM** ($n = 15$)
The rats were anesthetized and subjected to surgery except for NMDA injection. Sham rats were evenly divided into normal cage, immobilized wheel, and wheel groups. There were no significant differences observed in body weight or behavior tests among those groups, so they were pooled into one group.

**NMDA** ($n = 14$)
Rats were injected with NMDA and housed in a normal cage.

**W-24 h** ($n = 14$)
Rats were placed in a wheel cage nightly starting from night 1 post NMDA injection for a total of 14 nights.

**W-72 h** ($n = 14$)
Rats were placed in a wheel cage nightly starting from night 3 post NMDA injection for a total of 14 nights.

### 2.4. Behavioral measurements

Animals were pretested one day before surgery, and then again on days 1, 2, 7, 10, 14, 21, and 28 after the NMDA injection. All behavioral testing took place in a quiet and low-light room by an experimenter blinded with respect to the treatment groups. The following footfault, forelimb placing, and cylinder tests were done according to previously published methods [22].

#### 2.4.1. Forelimb placing

Animals were held by their torsos with forelimbs hanging freely. While one of the other limbs was gently restrained by putting the index finger of the examiner in front of the forelimb (see www.schallertlab.org for a movie), vibrissae-evoked placing of the other forelimb was examined. The rat was lowered horizontally until the contralateral or ipsilateral vibrissae contacted the upper edge of a tabletop. Ten trials were given. Percent successful placing responses were determined (number correct x 10) for contralateral and ipsilateral responses.

#### 2.4.2. Footfault

Animals were placed on an elevated grid, with openings of 2.3 $cm^2$, for 2 min. As the animals traversed the grid, a footfault was scored each time a paw slipped through an opening in the grid. The total number of steps was also counted. The percent footfault was calculated as the number of footfaults/total steps X 100 for ipsi- or contra- forelimb.

#### 2.4.3. Cylinder test

Animals were placed in a narrow glass cylinder (16.5 $\times$ 25 cm) and videotaped. The number of spontaneous movements made by each forelimb to initiate rearing, move laterally along the wall of the cylinder, or land on the floor after rearing were counted for 20 steps. The number of spontaneous movements of each forelimb was expressed as a percentage of the total to compute the "spontaneous limb use index" for each forelimb. This index has been used as a sensitive and reliable test of sensorimotor impairment and subsequent recovery following unilateral cerebral cortical injury [24,25].

### 2.5. Lesion volume measurement

Lesion volume and tissue atrophy were measured using H-E stained sections. After the last behavior tests on day 28, the rats were deeply anesthetized with choloral hydrate (0.6 g/kg) and intracardiac perfused with ice-cold PBS. The brains were removed and snap-frozen in 2-methylbutane on dry ice and stored in a $-80^{\circ}$C freezer. Coronal cryosections were cut using a Leica CM1800 cryostat, and 50 sections from bregma $+$ 2.5 mm to $-2.5$ mm were collected from each brain, 5 sections (20 $\mu$m thickness) every 0.5 mm. Sections were collected on Fisher Superfrost glass slides and dried on a 55°C Slide Warmer. Sections #1, #6, #11, #16, #21, #26, #31, #36, # 41, #46, and #50 were stained with H-E and used for lesion volume measurement (H-E stain). The other sections were used for immunohistochemistry.

The H-E stained sections were scanned into a Macintosh computer and the lesion volume was analyzed by a computer-interfaced BRAIN imaging system using the "indirect method" (area of the intact contralateral cortex – area of the intact ipsilateral cortex) to correct for brain shrinkage during processing [26]. Each hemisphere was measured separately by tracing with a cursor and transformed to $mm^2$. The lesion volume was computed by subtracting the NMDA lesioned (left) cortex from the intact (right) cortex on each section. The lesion volume for each rat was the sum from each section.

### 2.6. Immunohistochemistry

Coronal cryosections at bregma 1.0 mm (20 $\mu$m) were fixed in 95% methanol and 5% glacial acetic acid at $-15$°C for 10 min prior to immunostaining. The sections were washed and penetrated with 0.3% of NP-40 in PBS for 30 min at room temperature, then blocked with a blocking buffer (3% normal goat serum, 0.3% NP-40 in PBS) for 1 hour. The synaptophysin antibody (Sigma, Clone SVP-38) was diluted at 1:500 in the blocking buffer and incubated at room temperature for 2 hours. After brief washing, goat anti-mouse Ig was conjugated to Alexa-488 (Molecular Probes, Eugene, OR) at a dilution of 1:200 in the blocking buffer for one hour. After complete washing in PBS, the sections were coverslipped with VECTASHIELD mounting medium for fluorescence. The images were examined under a ZEISS AXIOSKOP 2 microscope with fluorescence power and captured using the connected CoolSnap Camera (Black-White Model) with imaging analyses by an IBM computer using the MetaMorph Imaging Series 6.1. The images were all acquired under the same conditions (light intensity, exposure time, magnification).

The average intensity of the immunofluorescence from regions of ipsilateral cortex lesion core, lesion border zone, adjacent corpus callosum, adjacent striatum, and contralateral cortex at the level of bregma $+1.0$ mm was measured in an area of 100 $\times$ 100 square nanometers. The average intensity from five sub-locations at each spot was used to calculate the fluorescence intensity of each sample. The mean and standard deviation of the average intensity from each group was calculated and used for data analysis.

### 2.7. Statistical analysis

Investigators blinded to the treatment assignment did all behavioral testing, and subsequent histological analyses. Data were expressed as mean $\pm$ SD and were analyzed by one-way ANOVA followed by Student-Newman-Keuls post hoc test, or two-way ANOVA with Bonferroni post-tests to compare replicate means at different time points.

## 3. Results

### 3.1. Wheel-running activity and body weight

We recorded the number of wheel-turns that were run voluntarily by each rat every night. There were no significant differences between the W-24 h and W-72 h animals, so we pooled the data from those two groups. Figure 1(B) shows the wheel-running number per rat per night during the 14 nights. The number of wheel-turns gradually increased from about 200 to 700 turns until the fifth night, and then reached a plateau which lasted for about 14 nights.

Body weight showed a temporary reduction in the first week after surgery, then gradually increased until day 28. There were no significant differences among the groups (data not shown).

### 3.2. Lesion Volume measurements

Figure 2(A) shows the lesion volume and location in the cortex on day 28. NMDA injection caused a cortical lesion about 24.01 $mm^3$. Wheel-running starting either from 24 or 72 hours did not have a significant effect on the lesion volume (23.04 $\pm$ 4.71 or 20.20 $\pm$ 7.9) compared to the NMDA control group ($p > 0.05$).

### 3.3. Behavioral measurements

#### 3.3.1. Forelimb placing

Figure 3 shows the behavior data of each group on forelimb placing in response to vibrissae stimulation. Sham surgery induced only a temporary and slightly abnormal forelimb placing score on day 1, which had recovered to baseline by the next day. NMDA injection to the forelimb sensorimotor cortex induced significant forelimb-placing deficits on days 1, 2, 5, and 7. These deficits recovered to sham surgery levels by day 10 and were completely back to normal by day 14. Wheel-running starting on day 1(W-24 h) tem-

Table 1
Significance in forelimb placing

| | Days after NMDA injection | | | | | | | |
|---|---|---|---|---|---|---|---|---|
| | 1 | 2 | 5 | 7 | 10 | 14 | 21 | 28 |
| NMDA to sham | *** | *** | ** | * | ns | ns | ns | ns |
| NMDA to W-24 h | ns | ns | *** | ns | ns | ns | ns | ns |
| NMDA to W-72 h | ns | ns | ns | ns | ns | ns | ns | ns |
| W-24 h to Sham | *** | *** | *** | * | ns | ns | ns | ns |
| W-72 h to Sham | *** | *** | ** | ns | ns | ns | ns | ns |
| W-24 h to W-72 h | ns | ns | *** | ns | ns | ns | ns | ns |

Whisker placing data was analysed by two-way ANOVA and Bonferroni post-tests to compare replicate means at different timepoints. "ns", $p > 0.05$; "*", $p \leqslant 0.05$; "**", $p \leqslant 0.01$; "***", $p \leqslant 0.001$.

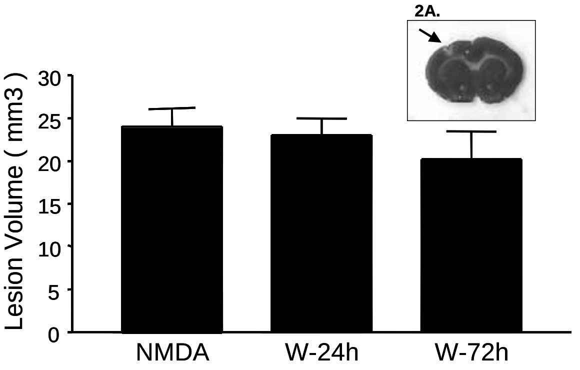

Fig. 2. Lesion Volume measured on day 28 after NMDA lesion. The data are expressed as mean ± SD. The insert (2A) is a photo showing the lesion location in the cortex. The light-stained area in the cortex is the location of NMDA lesion.

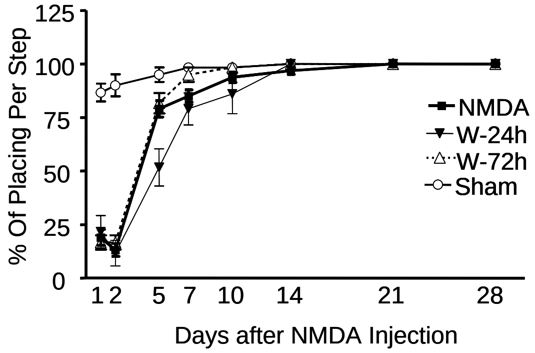

Fig. 3. Whisker-placing index of the contralateral forelimb. The data are expressed as mean ± SD.

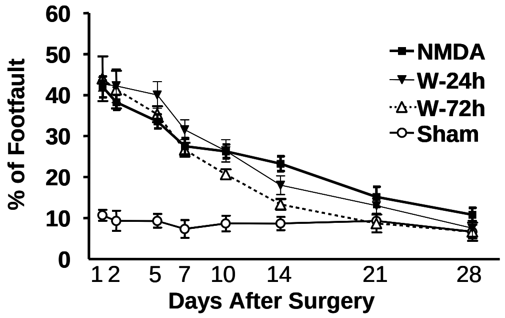

Fig. 4. Percent of footfaults in the contralateral forelimb. The data are expressed as mean ± SD.

porarily worsened forelimb placing on day 5, but there was no difference from the NMDA group by day 7. Wheel-running starting from day 3 (W-72 h) significantly speeded recovery of forelimb placing by day 7, 3 days earlier than the NMDA alone group. However, although the W-72 h group recovered faster than the NMDA alone group when both groups were compared to controls, at no time did the two groups significantly differ when compared against each other. The results of Bonferroni post-tests to compare replicate means of vibrissae-stimulated forelimb placing at different time points are shown in Table 1.

### *3.3.2. Footfault*

The footfault data show the same patterns as the placing test data except that recovery was much slower, and the positive effect of delayed wheel-running was more apparent (see Fig. 4). NMDA injection caused significant footfaults starting on day 1 through day 21, and the rats did not recover to sham level until day 28. Again, wheel-running starting from day 1 (W-24 h) temporarily worsened performance on days 5 and 7 compared to the NMDA group. The wheel-running group starting from day 3 (W-72 h) showed recovery on day 14, one week earlier than the control group. The W-72 h group performed significantly better than the NMDA alone group at all time points after 10 days. The results of Bonferroni post-tests to compare replicate means of Footfault at different time points are shown in Table 2.

Table 2
Significance in footfault

| | Days after NMDA injection | | | | | | | |
|---|---|---|---|---|---|---|---|---|
| | 1 | 2 | 5 | 7 | 10 | 14 | 21 | 28 |
| NMDA to sham | *** | *** | *** | *** | *** | *** | * | ns |
| NMDA to W-24 h | ns | ns | * | * | ns | ns | ns | ns |
| NMDA to W-72 h | ns | ns | * | ns | ns | * | * | * |
| W-24 h to sham | *** | *** | *** | *** | *** | * | * | ns |
| W-72 h to sham | *** | *** | *** | *** | ** | * | ns | ns |
| W-24 h to W-72 h | ns | ns | ns | ns | ns | ns | ns | ns |

Footfault data was analysed by two-way ANOVA and Bonferroni post-tests to compare replicate means at different timepoints. "ns", $p > 0.05$; "*", $p \leqslant 0.05$; "**", $p \leqslant 0.01$; "***", $p \leqslant 0.001$.

Table 3
Significance in simultaneous use of both forelimbs in cylinder test

| | Days after NMDA injection | | | | | | | |
|---|---|---|---|---|---|---|---|---|
| | 1 | 2 | 5 | 7 | 10 | 14 | 21 | 28 |
| NMDA to sham | *** | *** | *** | *** | *** | *** | ** | * |
| NMDA to W-24 h | ns | ns | ns | ns | ns | ns | ns | ns |
| NMDA to W-72 h | ns | ns | ns | ns | ns | ns | * | ** |
| W-24 h to sham | *** | *** | *** | *** | *** | *** | *** | ** |
| W-72 h to sham | *** | *** | *** | *** | *** | ** | ns | ns |
| W-24 h to W-72 h | ns | ns | ns | ns | ns | ns | * | ** |

Simultaneous use of both forelimbs in cylinder test data was analysed by two-way ANOVA and Bonferroni post-tests to compare replicate means at different timepoints. "ns", $p > 0.05$; "*", $p \leqslant 0.05$; "**", $p \leqslant 0.01$; "***", $p \leqslant 0.001$.

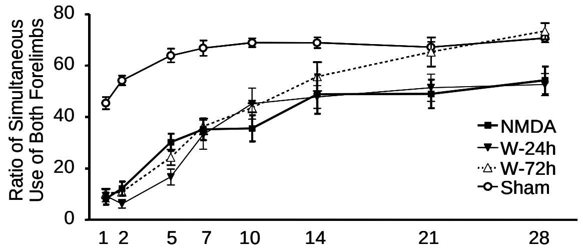

Fig. 5. Ratio of simultaneous use of both forelimbs in Cylinder test. The data are expressed as mean ± SD.

#### 3.3.3. *Spontaneous limb-use*

We also saw a significant difference between groups on simultaneous use of both forelimbs during vertical-lateral exploration of the walls of a cylindrical enclosure (see Fig. 5). More than 50% of the time, normal rats typically exhibit simultaneous movement of both forelimbs to support/brace themselves or step laterally along the wall of the cylinder, and to land on the floor after rearing. Sham rats showed only a temporary decrease of simultaneous forelimb use on days 1 and 2, and returned back to baseline level on day 7. The NMDA lesion caused a significant decrease in the simultaneous use of both forelimbs. While this deficit improved over the month of observation, it had not returned to sham levels by the end of the experiment on day 28. W-72 h significantly promoted the recovery of simultaneous forelimb use, returning to sham levels by 21 days. Delayed wheel-running (W-72 h) resulted in faster recovery compared to the NMDA alone group after 14 days. The results of Bonferroni post-tests to compare replicate means of simultaneous use of both forelimbs at different time points are shown in Table 3.

### 3.4. *Synaptophysin*

We examined brains to see if the behavioral recovery was associated with synaptophysin immunoreactivity. In the lesion core, we observed a pronounced loss of synaptophysin immunoreactivity on day 28 after the NMDA lesion (Fig. 6A). Increased synaptophysin immunoreactivity was observed in the zone adjacent to the lesion core in the cortex, corpus callosum, and ipsilateral striatum and contralateral cortex in all groups (Fig. 6B, C, D, E, and F). Quantitation of synaptophysin immunoreactivity detected some differences between groups at 28 days after surgery (Fig. 7). Synaptophysin immunoreactivity in the W-72 h group was denser than in the NMDA control group, especially in ipsilateral lesion border zone, corpus callosum, and striatum and contralateral cortex, but these differences were not significant ($p > 0.05$).

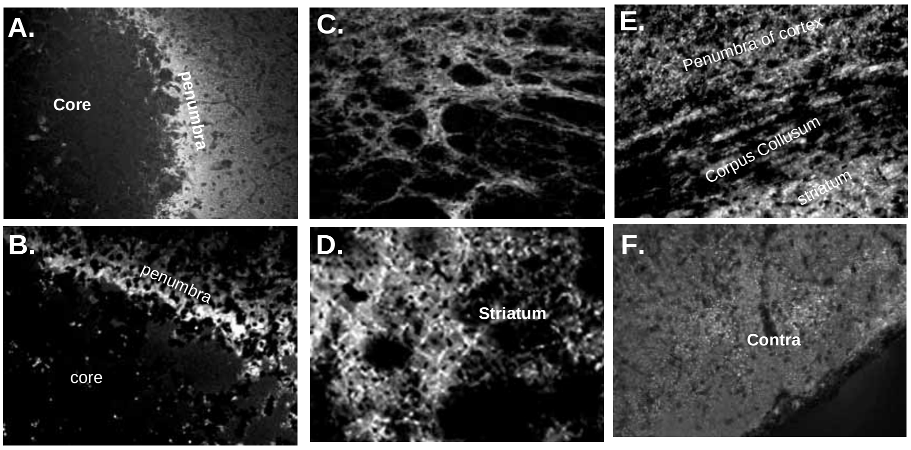

Fig. 6. Photograph of synaptophysin immunofluorescence. The coronal section at bregma +1.0 mm were immunostained with synaptophysin and photographed from ipsilateral cortex (A, 10x), mirror-reflected contralateral cortex (B, 10x), ipsilateral striatum adjacent to the lesion (C, 10x), and amplified photos from ipsilateral cortex (D. 20x), corpus callosum (E, 20x), ipsilateral striatum (F, 20x).

## 4. Discussion

We found in this NMDA cortical lesion model that wheel-running, initiated after 24 h or 72 h and lasting for two weeks, did not influence lesion volume detectably. Therefore, unlike some previous studies from our own lab and others [18–20,27], we did not find that early exercise therapy in this model was detrimental. However, we did detect a temporary impairment of motor recovery 5–7 days after the insult if wheel-running was started early (i.e. at 24 hours after the lesion).

We found that wheel-running delayed until 72 h after the cortical lesion had a significant effect on speeding the early re-acquirement of sensorimotor neurological function as measured by behavior tests including whisker-stimulated forelimb placing, footfault, and spontaneous limb use. However, earlier wheel-running initiated 24 h post lesion did not positively affect recovery. While delayed wheel-running speeded recovery to normal levels on all 3 tests, the differences in recovery between trained (W-72 h) and untrained (NMDA) lesioned rats did not reach significance on the forelimb placing test. On the footfault and cylinder tests, better recovery in trained animals compared to untrained could be readily seen after 2 and 3 weeks respectively, and persisted until the end of the experiment at 1 month. We do not have an explanation for the lesser effect on the forelimb placing test. It may be that this test is less sensitive to the beneficial biological effect of exercise training.

The underlying mechanisms for such effects remain unclear. We found somewhat more intense synaptophysin immunoreactivity in the border zone of cortex around the lesion, the corpus callosum directly underneath the lesion, and the ipsilateral striatum. Although whisker-stimulated forelimb placing scores had returned to sham levels by 7 days, one cannot expect that the timing of markers associated with plasticity and behavioral improvements would overlap necessarily. Wheel-running generally upregulates trophic factor expression and related mechanisms associated with plasticity in the brain [28]. Promotion of early recovery by wheel running could be related to prevention of remote degeneration of tissue, but also might be linked to adoption of alternative behavioral strategies to compensate for impaired performance.

Focal neocortical infarction creates a circumscribed lesion in the neocortex that may provide a denervation-related stimulus for neurite growth and perhaps even synaptogenesis. In the period after cerebral ischemic infarction, massive neuronal death results in regions of denervation, which could provide a stimulus for undamaged neurons to sprout and establish new synaptic connections. Synaptophysin upregulation in regions around the infarct core in our experiments suggests that considerable neuronal remodeling and plasticity may have occurred in the peri-injury area after cerebral infarction. Recovery after stroke largely depends on widespread functional and structural neuronal plasticity. Advances in neuro-functional imaging methods such as Positron Emission Tomography (PET), Magnetic Resonance Imaging (fMRI), and Transcranial Magnetic Stimulation (TMS), which permit visualization of training-induced plasticity in stroke patients, have suggested that rehabilitative interventions have benefits by modulating neuro-plastic reorganiza-

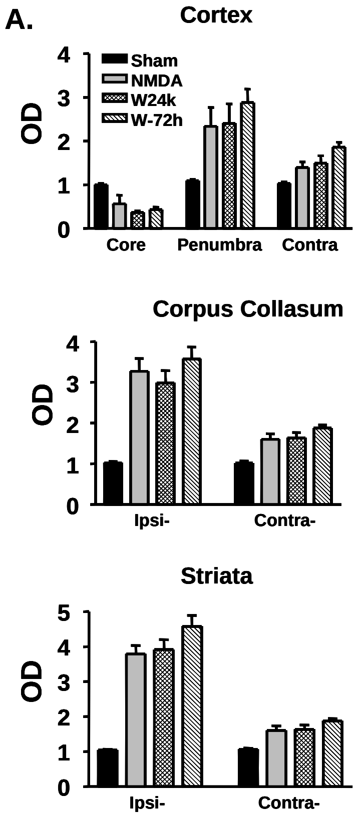

Fig. 7. Quantitation of synaptophysin immunofluorescence in cortex (A), corpus collasum (B) and striatum (C). The data are expressed as mean ± SD. The average intensity of the sham group is expressed as OD of 1.0, other groups are normalized to the sham group.

tion in stroke patients. These discoveries have important implications for the future development of neurorehabilitative training methods. Timing of therapy and drug delivery appear crucial; the optimal "critical period" has not yet been clearly identified [29,30]. Our study may provide a hint of the optimal time window for rehabilitative interventions in clinical practice.

## 5. Conclusion

Wheel-running speeds neurological recovery when started 72 h, but not 1 day, after an excitotoxic insult. Further studies are needed to elucidate mechanisms responsible for enhancing neurite sprouting and synaptogenesis, and to evaluate the interaction of physical and pharmacologic therapies targeting brain recovery and repair.

## Acknowledgments

This work was supported by grants from James C. Grotta, MD (R01 NS23979), and Jaroslaw Aronowski, PhD (R01 NS039378). We also thank Gabriela Redwine for her editorial help.

We also appreciate the suggestions of Harvey Levin PhD, who kindly reviewed the manuscript.
